# Supporting Social Inclusion in Neighbourhoods of Adults with Intellectual Disabilities: Service Providers’ Practice Experiences

**DOI:** 10.1177/17446295221085479

**Published:** 2022-04-21

**Authors:** Geraldine Boland, Suzanne Guerin

**Affiliations:** UCD Centre for Disability Studies, UCD School of Psychology, 8797 University College Dublin, Dublin, Ireland; UCD Centre for Disability Studies, UCD School of Psychology, 8797 University College Dublin, Dublin, Ireland

**Keywords:** intellectual disability, community participation, social inclusion, service provision, neighbourhood

## Abstract

Deinstitutionalisation has increased the likelihood of adults with intellectual disabilities residing in neighbourhoods either in staff-supported accommodation or in their family home. However, it raises the question of whether national policies on disability have translated into practice actions by service providers that result in positive social inclusion outcomes for individuals. This study examined the practice initiatives supporting social inclusion in neighbourhoods in specialist state-funded service providers for adults with intellectual disabilities. Using a mixed methods design, CEOs/service leaders of 40 organisations completed an online survey. Follow-up interviews were completed with a randomised sample. Shifting towards new service models and strategic links with mainstream organisations were most often mentioned as furthering social inclusion goals. A wide range of service initiatives were reported, with positive outcomes alongside a range of challenges. Service providers play an important role in providing individualised supports that foster local engagement. However, the service context is complex and service leaders have reported many challenges that may impede progress on social inclusion.

## Introduction

Since the 1980s the movement towards closing institutions and developing community-based services has been a dominant force in the intellectual disability service context in the developed world and an impetus for applied research in the sector (Parmenter, 2004). The UN Convention on the Rights of People with Disabilities (UNCPRD) proposed a guiding principle of full and effective participation and inclusion in society for all people with disabilities. The risk of social exclusion and isolation for people with disabilities is underscored by the proposal: ‘that persons with disabilities have access to a range of in-home, residential and other community support services, including personal assistance necessary to support living and inclusion in the community and to prevent isolation or segregation from the community’ (UNCRPD, 2006, article 19). The EU Fundamental Rights Agency (2017) tracked progress on deinstitutionalisation across Europe, focused on indicators of the UNCRPD (2006) Article 19. While the pace of deinstitutionalisation has differed, cross-European comparisons are hampered by lack of consistent comparable data. However, they concluded that, for people with all disability types, lack of appropriate support services, impediments to accessing mainstream services and narrow choices about where to live, restricted equal community participation of Europeans with disabilities. Internationally, many countries that have ratified the UNCRPD have initiated new, or further developed existing national strategic disability plans that explicitly identify local participation, (e.g. New Zealand (Government of New Zealand, 2016); Victoria state, Australia (State of Victoria, Australia, 2016) and the UK (UK Government, 2009)). However, how the aspiration of full participation may be achieved in practice was not specified. In the case of the UK Valuing People policy, the impact of austerity, with significant cuts to local authority funding has led to a loss of momentum on social inclusion, thus undermining progress on the overall policy objectives (Power and Bartlett, 2018, 2019).

Social relationships, inclusion and self-determination are deemed particularly threatened, for any person with intellectual disability residing in a hospital or segregated cluster-housing scheme (Mansell and Beadle-Brown, 2012). Living locally is understood as residing in dispersed housing, that is: ‘apartments and houses of the same types and size that the majority of the population live in, scattered throughout residential neighbourhoods, among the rest of the population’ (Mansell and Beadle-Brown, 2009, p.314). Dispersed housing also includes an adult with intellectual disability continuing to live with his/her family or adoptive/foster family. However, residing in neighbourhoods does not always equate with social engagement, forming new relationships or having a sense of connection to place. Social exclusion may be understood to relate to persistently high levels of stigma globally towards people with intellectual disabilities (Scior et al., 2020) and review evidence that they are perceived by some non-disabled people as ‘highly undesirable for social interactions’ (Scior, 2011, p.2178). Despite being offered locally based services, people with high support needs are found to be most at risk of relationships limited to residents they live with (not necessarily of their choosing) or paid staff (Mansell and Beadle-Brown, 2012). Irrespective of accommodation type, loneliness is a common experience for this population, in particular those over 40 years who depend on staff support (Wormald et al., 2019). Without the awareness required to recognise and overcome barriers to inclusion, an adult with intellectual disability risks being invisible in their locality, or enduringly perceived as a client under supervision or an object of pity (O’Brien and Mount, 2015). A consensus is evident in reviews on social inclusion that to attain positive life benefits, residing in the mainstream of society and actively participating is desirable (e.g. Bigby, 2012; Overmars-Marx et al., 2014). Whether people live in staff-supported services or in a family home, service providers have resources and scope to support individuals to move beyond being physically present in localities to social inclusion that leads to valued engagement.

Adults with intellectual disabilities engaging outside their homes has been variously titled community participation, community integration, social participation and more recently social inclusion. Reviews acknowledge community participation as an unclear (e.g. Amado et al., 2013; Bigby, 2012) contested concept (Clifford-Simplican, 2019). The following definition is derived from the prior literature (e.g. Bigby and Wiesel, 2019; Cobigo et al., 2012):

Social inclusion in a neighbourhood for individuals is defined as active engagement with people and places that matter to an adult with intellectual disability based on their individual preferences, in the immediate locality in which they live. This engagement may be achieved by being supported to become known by sight or by name in their neighbourhood; engage in valued social roles; have equal access to public goods and services; and belong to a growing network of connections which may include family, mutually supportive neighbours, acquaintances and friendships. The experience of being socially included in their neighbourhood for a person with intellectual disability is not static. With support to engage in convivial encounters, interactions and new experiences, identification with and attachment to place, with a sense of belonging may develop for the individual (Boland and Guerin, 2021, p.2).

The influence of national disability policies on service providers policies and practice has received limited attention in the intellectual disability literature. Policy makers and service providers were found to be engaged in a time consuming and slow process (Power, 2013), with barriers including – the benefits trap negatively impacting employment, cutbacks to services, labour intensive accountability systems, service safety policies/curfew rules and scarcity of dedicated resources to mobilise individualised approaches (Simplican et al., 2015). In the context of the present study, the specific influences, if any, of the UNCRPD (2006) and national disability policies on service providers’ practice interventions to support social inclusion in neighbourhoods was unknown. Irish policy proposed the closure of congregate residential settings (Health Service Executive [HSE], 2011) and an active national implementation plan was underway at the time of the present study (HSE, 2016). However, a trend of refilling vacant places in these settings was confirmed in a longitudinal study using the national Irish intellectual disability database, signalling the undermining of this policy by some service providers (McConkey et al., 2018). In tandem, implementation of a policy titled New Directions on the reform of traditional segregated day services was underway, with an expectation that Irish service providers would forge engagement in localities for adults with intellectual disabilities (HSE, 2012). Ireland was identified as policy rich; however, the challenge of implementation had been recognised (Linehan et al., 2014).

While the role of direct support staff in supporting social inclusion has been explored empirically (e.g. (Bigby et al., 2012); McConkey and Collins, 2010), a recent systematic review of literature on social inclusion (Boland et al., in press) highlighted that the perspectives and practice experiences of those with overall leadership responsibility for supporting social inclusion has received limited attention (e.g. Power, 2013). No study was traced that exclusively examined the practice interventions to support social inclusion in neighbourhoods across a range of service organisations serving different geographic areas. The aim of this study then, was to conduct a national study across the full range of service provider types. The intention was to inform an understanding of how specific policy goals on social inclusion in localities can be best realised in service settings, with consideration of both the opportunities and challenges, the role of direct support staff and the role of families.

This study aimed to examine the practice experiences of service provider organisations as perceived by their CEOs/service leaders in supporting social inclusion in neighbourhoods of adults with intellectual disabilities, asking:

What actions (if any) have been tried, or interventions (if any) tested, to support adults with intellectual disabilities living in dispersed housing to participate in their neighbourhoods?

## Method

### Research design

An explanatory sequential mixed methods design (Creswell and Plano-Clarke, 2018) was developed to capture indicative examples of service providers’ supports for social inclusion. As conceptualised by Creswell (2015), this design begins with a quantitative strand, with a second qualitative strand to explore the quantitative results. Mixed methods allows researchers to gather rich data that serves to answer complex research questions (Creswell and Plano-Clark, 2018) such as an exploration of initiatives to support social inclusion. An online survey included demographic and topic-based questions to explore practice experiences (Braun et al., 2020), while qualitative semi-structured telephone interviews (15%, n=6) with a maximal variation random sample (Creswell, 2015) gathered more in-depth responses. Telephone interviews were chosen for convenience of service leaders, lasting between 23 to 42 minutes.

### Participants and sampling

The target population for this study was CEOs (or comparable senior staff) of all Republic of Ireland organisations providing specialist services to adults with intellectual disabilities living in their family homes or in dispersed staff-supported housing (with 62 such organisations identified). The sample did not include mainstream community services serving the general population that also include people with disabilities. Neither did it include housing associations/services that serve marginalised groups, as in Ireland housing and accommodation needs of adults with intellectual disabilities are met in the main by specialist intellectual disability service providers. The organisations were sampled from across the Irish intellectual disability specialist services sector including – voluntary bodies receiving full state funding but also holding charitable status (n=49, 79.03%), services directly funded and managed by the Health Service Executive (HSE) (n=8, 12.90%) and private companies also receiving funding from the HSE (n=5, 8.06%). All services are bound by relevant national policies (e.g. New Directions (Health Service Executive HSE, 2015)) and regulatory requirements for staffed residential services (Health Information and Quality Authority, 2013).

In identifying the target for invitations, a range of senior roles were considered in addition to CEOs for a number of reasons. Some organisations did not have a CEO, while in larger operations the CEO may not be involved in a way that would facilitate completion of the survey/interview. For consistency, the CEO (or equivalent title) of each of the 62 organisations was invited to participate, with the option to recommend a senior colleague in their place. A total of 42 individuals consented (67.72% response rate), with a survey completion rate of 64.50% (n=40). The reach of the survey was strong, with representatives of services in 25 of 26 counties in the Republic of Ireland returning a single survey for each organisation. All three types of organisations were represented, with the largest group coming from voluntary bodies (77.5%, n=31) (see Table 1 for organisational information and Table 2 for individual demographics). In addition, there was good variation in the size of services and their geographic locations. Participating leaders were predominantly in management roles, with some variation in the length of time in their current role. However, the majority had been with the organisation for more than 10 years. While a large proportion of participants agreed to take part in interviews, six were selected to ensure broad representation of the group, with five completing scheduled interviews.

**Table 1.** Service Provider Type, Size and Types of Geographic Locations Served.

		
*Service provider type of organisation*	*n=40*	*100%*
Voluntary Service Providers^a^	31	77.5
HSE directly managed services and private providers^b^	9	22.5
*Service provider size-number of adults with intellectual disabilities served*	*n=40*	*100%*
Fewer than 200 adults	20	50
Between 200 and 1500 adults	17	42.5
Over 1500 adults	3	7.5
*Types of geographic locations served*	*n=40*	*100%*
Town only	6	15
City/suburbs only	6	15
Town, rural village and/or remote rural	7	17.5
City suburbs, town and/or village	5	12.5
All location types	16	40

^a^Both types of voluntary service providers (designated Section 38 and Section 39, Health Act, 2007) have been combined to protect confidentiality.

^b^These two categories have been combined to protect confidentiality.

**Table 2.** Service Leaders Job Titles and Length of Service.

	N	%
*Job title cluster*	*40*	*100*
Management roles	29	72.5
Clinical leadership roles	5	12.5
Social inclusion leadership roles	6	15
*Length of service in current role*	*40*	*100*
Less than 5 years	18	45
Between 5 and 10 years	4	10
Between 10 and 15 years	6	15
Between 15 and 20 years	9	22.5
20 plus years	3	7.5
*Total length of service with current organisation*	*40*	*100*
Less than 5 years	8	20
Between 5 and 10 years	1	2.5
Between 10 and 15 years	14	35
Between 15 and 20 years	9	22.5
20 plus years	8	20

## Materials

A mixed methods survey containing both qualitative and quantitative questions was designed to gather the key information required to address the research questions. The survey included closed and open questions, with closed questions using multiple choice and five-point Likert scale responses. The question order was informed by guidelines developed by Dillman and Edwards (2016). Open-ended questions were included with scope for survey respondents to include free text responses to capture their practice experiences. For example, one survey question offered structured prompts for respondents to describe in detail an indicative practice initiative aimed at supporting social inclusion including: objectives, a summary description, challenges, if any and the outcome. An important consideration in the design of the survey was maximising engagement and minimising time required to complete the survey. The interview, completed with a subsample of participants, was designed to explore responses to the survey in more detail on service initiatives summarised in the survey responses. Examples of areas probed included the role of staff and families in supporting social inclusion.

### Procedure

The survey was designed for delivery using the Qualtrics Research Core platform (https://www.qualtrics.com/research-core/). Development included pretesting with four stakeholders, including two retired CEOs. Invitations to participate were sent directly to the CEOs/leaders in the 62 organisations included in the sampling frame, with two follow-up calls used as reminders. The survey was also promoted by the National Federation of Voluntary Service Providers to its member organisations and highlighted in social media. Ethical approval for the study was secured from the host academic institution (University College Dublin HS-18-75-Boland-Guerin) and from service provider organisations where required. All participants returned a signed consent form prior to completing the survey. Telephone interviewees returned an additional consent form. Service organisations are not named for confidentiality reasons.

### Data analysis

The data comprise both quantitative and qualitative information. Responses to open-ended questions were analysed using an inductive content analysis (Hsieh and Shannon, 2005; Mayring, 2000), while quantitative data were analysed using frequency counts, means and standard deviations. Given the mixed nature of the data, a range of techniques were employed to ensure the validity of the analysis and the resulting findings, including formal credibility checks and a reflective approach to interpretation. The analysis was completed by three researchers with strong experience of either the disability sector or qualitative analysis. They worked independently with the data, before coming together to discuss observations on the data, which ultimately informed the lead researcher in their interpretation. The inductive content analysis approach allowed the more conceptual narrative content of survey responses and interview transcripts to be coded, clustered and categorised. The sequence of steps followed in this qualitative analysis are included in Supplemental Material 1.

## Results

Almost all survey respondents (n=37, 92.5%) reported that their organisation had been involved in initiatives aimed at supporting adults with intellectual disabilities to participate in their neighbourhood. Respondents were prompted to give details of one of the actions or interventions that their organisation had undertaken at an individual or group level, an initiative that had a desired result or one that they deemed to be not so successful. Three types of service initiative were discernible:

• a service initiative focused on one individual with intellectual disability
• a service initiative focused on a group of between two and 20 adults, or
• an organisation-wide service initiative potentially reaching more than 20 people with intellectual disabilities.

The practice examples from both surveys and interviews are summarised in Table 3, with a total of 68 initiatives identified. The breakdown of examples identified by source is also reported. As Table 3 highlights, there was a relatively equal spread of the three types of service initiative summarised by survey respondents, from actions to support individuals, group initiatives, with slightly less organisation-wide projects.

**Table 3.** Service Initiative Types Supporting Social Inclusion in Neighbourhoods of Adults with intellectual disabilities.

Types of service initiative	Service initiative examples from survey responses only (n=40)	% of total responses (both survey and telephone interviews)	Service initiative examples from telephone interview responses (n=28)	% of total responses (both survey and telephone interviews)	Service initiatives total (n= 68)	Total % 100%
Service initiatives focused on an individual	13	19.12	11	16.8	24	35.29
Service initiatives focused on two to 20 adults	13	19.12	5	7.35	18	26.47
Organisation-wide service initiatives	11	16.18	12	17.65	23	33.82
No service initiative reported by service provider	3	4.41	0	0	3	4.41

### Service initiatives focused on individuals

For initiatives focused on an individual, survey respondents summarised a range of activities supporting social inclusion locally through employment, volunteering, and people being supported to pursue solo leisure interests, for example, art. The range of examples also included getting opportunities to sample new activity or skills-based leisure groups to support choice making for individuals, based on their preferred level of involvement. The following is a description of a service initiative focused on one individual involving a range of support people, with the focus on personal goals related to health, fitness and local engagement (see Box 1). Challenges noted were transport and the flexibility of day service staff to work during evening hours.

Box 1. Service Initiative Example Focused on an Individual

**Table:** 

*Objective:* ‘To support a person to achieve their goal of weight loss and improved health and fitness.’ *Describe project/action:* ‘The person specified that they would like to lose weight, become fitter and more engaged with their local community.’ *Who was involved?* ‘The person with an intellectual disability, their key worker, the slimming club, the leader of the local walking group.’	*Challenges faced:* ‘The walking club met at night-time and transport was initially a challenge. The keyworker was required to work in the evening (we are traditionally a day service) to bring the person on a couple of walks with the group to determine if it was suitable.’ *Outcome:* ‘The person is now an active member of the weekly walking club. The keyworker found a person in the club with a social conscience who now collects the person to take them to the walking start point. This allowed the keyworker to withdraw from the walking group and return to normal working hours. The person has lost several stones in weight and attends the walking club’s social events. The person nominated the person in the group for a [*Title of award and date*].’

### Service initiatives focused on groups

Survey responses on initiatives for groups of 2 to 20 adults with intellectual disabilities were diverse, with examples including a self-advocacy group focused on local political action to change the location of a local bus stop and new group home residents getting to know their neighbours through staff supporting them to host a house-warming party. The following example is of a group of men with intellectual disabilities who became volunteer members of a local clean-up group, with the challenge of maintaining motivation highlighted, in addition to the benefits of developing new connections that extended beyond the shared volunteer activity (see Box 2).

Box 2. Service Initiative Example Focused on a Group of Adults

**Table:** 

*Objective:* ‘Membership of the local tidy towns committee.’ *Describe project/action:* ‘A group of young men joined the local tidy towns initiative and actively participated in the clean ups, they also had areas of responsibility for keeping their area clean and making and providing hanging baskets for the community.’ *Who was involved?* ‘Volunteers from the tidy town committee.’	*Challenges faced:* ‘Each person had to sign and commit to undertaking the work involved, this sometimes proved difficult to keep them motivated to carry out on their duties as part of the team.’ *Outcome:* ‘The outcome was that the men made connections with people in their community, they made a positive contribution. They also benefited from friendships where individuals took some of them to football matches.’

### Organisation-wide initiatives supporting social inclusion in neighbourhoods

Organisation-wide initiatives included the development of new service models supporting inclusion in localities, in the context of national policies and the move away from traditional campus-based services. One participant highlighted the role of supported living arrangements in setting the foundation for individuals to engage with greater autonomy in their neighbourhood. Organisation-wide initiatives involving the opening of new service locations as springboards to employment, education and neighbourhood involvement were offered, with an example summarised in Box 3. This highlights that family members are also stakeholders in these change initiatives. The importance was stressed of locating service buildings centrally, so that most engagement opportunities were within walking distance. Relationships needed to be forged in new localities to source social inclusion opportunities.

Box 3. Service Initiative Example at Organisational Level

**Table:** 

*Objective:* ‘Support people to attain valued roles in their community.’ *Describe project/action:* ‘The project involved securing specific town locations where people could be linked to their community and neighbourhoods. Specific project to support people to develop their image and competencies to actively participate.’ *Who was involved?* ‘Day service team, families and people we support.’	*Challenges faced:* 1. ‘Securing funding 2. Securing suitable locations with access etc. 3. Managing risk and safeguarding 4. Building relationships 5. Resistance to change’ *Outcome:* ‘Positive outcome with [*number of*] new locations opened and many people using same as a springboard to employment, education and involvement in their communities. Positive behaviour change and higher expectations from families.’

One service provider supporting people in both town and rural locations described a well-established outreach service.

*‘We do have a very strong outreach team now…service users don’t attend our services as such. They are supported by outreach workers. So a lot of those people are very individual, but they might be, that might be only 15 hours a week, but that’s very successful in terms of really helping people to be supported in their actual neighbourhood, in their actual locality, because they are not travelling to a centre for the day.’* (ID08, telephone interviewee)

### Positive outcomes for individuals and neighbourhoods/localities associated with service initiatives

**Figure 1.**
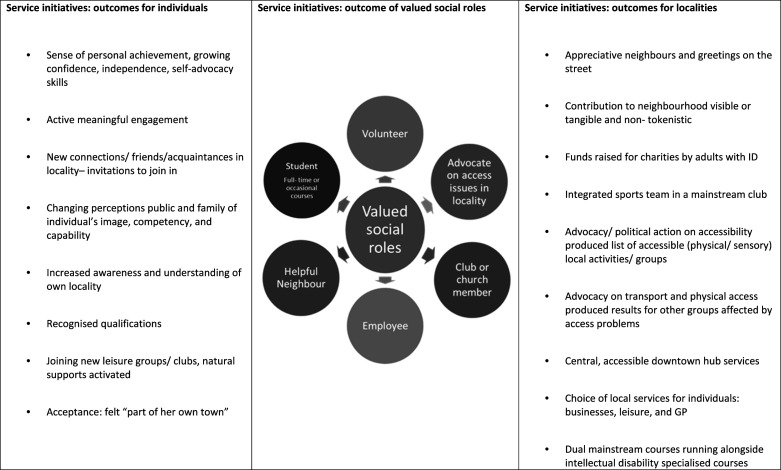
Service Initiatives Supporting Social Inclusion in Neighbourhoods – Multiple Diverse Outcomes.

Multiple diverse outcomes were reported for the service initiatives outlined by participants. As indicated in Figure 1, these included positive personal outcomes for individuals with intellectual disabilities, valued social roles, in addition to positive outcomes of service initiatives for the localities in which adults with intellectual disabilities live. Securing valued social roles was an important outcome from service initiatives. While positive outcomes for individuals was the objective of service initiatives, the positive contributions of adults with intellectual disabilities to their localities was also noted and is presented in Figure 1.

For individuals with intellectual disabilities, positive personal outcomes included growing independence and confidence based on personal achievement (e.g. acquiring a qualification or securing a job). One service provider described a year-long course for 20 adults with intellectual disabilities aimed at developing knowledge about where they live, noting an outcome of ‘increased awareness and understanding of one’s own community’ (ID35, survey response). Active engagement in local leisure groups/clubs was described as bringing opportunities to become known and to develop new acquaintances and in some cases friendships. Some service leaders commented on changing public and family perceptions of the image, competency and capability of the people with intellectual disabilities they supported. Public recognition as an adult with agency and developing self-advocacy skills were also positive outcomes. One woman supported to choose a general practitioner, explore leisure options, and shop locally, was reported to have said that she now ‘felt part of her own town’ (ID40, survey response).

A particularly relevant outcome was valued social roles. As seen in Figure 1, the range of social roles varies considerably from the relatively passive role of church member to becoming a proactive advocate on local access issues. Service initiatives also resulted in positive outcomes for localities based on contributions of adults with intellectual disabilities. As detailed in Figure 1 survey respondents reported a range of outcomes, from adults with intellectual disabilities contributing to local charities, to forming an integrated team in a mainstream sports club. Visible/tangible contributions to neighbourhoods were highlighted, including an individual (described as having high support needs and on the autistic spectrum) supported as a volunteer to tidy up and maintain an important public space in the lead up to a popular local event. It was reported that this ‘reinforced his foothold in this rural community and the sense that he was somebody of capacity and with a contribution to make’ (ID37 survey response). Calling for civic improvements was also noted, for example, adults with intellectual disabilities supported to advocate on poor transport or physical access were valued contributions to other groups also affected by access constraints. The survey respondents who offered a summary account of service initiatives (92.5%, n=37) were asked to rate their overall satisfaction with the outcome they had reported whether for an individual, a group of adults or an organisation-wide initiative. Thirty-one respondents (83.78%) were very satisfied or satisfied, with the remaining ranging from neither satisfied nor dissatisfied (5.4%, n=2) to dissatisfied or very dissatisfied (10.82%, n=4).

### Challenges associated with service initiatives

While most service leaders were either satisfied or very satisfied with the single service initiative they had summarised, they outlined many challenges. Participants tended to offer more narrative detail about the challenges than for the positive outcomes listed above. From content analysis, four categories of challenges were identified including:

• concerns about negative attitudes and preconceptions of locals
• individual characteristics of adults with intellectual disabilities with potential to negatively impact on their engagement
• managing changes to new locally based service models, and
• staff lacking the skills to facilitate social inclusion in localities and/or inadequate staffing levels.

Within each of these, clusters of specific challenges are detailed in Table 4.

**Table 4.** Service Initiatives Supporting Social Inclusion in Neighbourhoods – the Challenges Reported.

Category	Clusters of challenges	Sample quotes
Concerns about negative attitudes and preconceptions of locals	1. Challenge of building relationships 2. Lack of natural supports 3. Tokenistic club membership or different treatment 4. Near neighbours- worry about house values and potential disruption	*‘We tend not to have many challenges in acceptance, but we do have challenges in support for to get meaningful participation. And we also have challenges, it’s a constant challenge to try and get the right job…. Its people developing into more meaningful things and developing the social networks related to that. So you go to get a job to have a meaningful occupation and to have a valued role and to get paid, but you also do it to build relationships, not necessarily particularly strong relationships, but a group of friends and sometimes we struggle with that’* (ID08, telephone interviewee).
Individual characteristics of adults with intellectual disabilities that may negatively impact on their engagement	1. Challenging health care needs and ill-health 2. Social, communication and decision-making skills to be learned 3. Behaviours of concern 4. Fear of new experiences 5. Motivation and commitment-inconsistent attendance 6. Individual challenges faced by adults on the autistic spectrum with intellectual disabilities	One service leader clarified the need for intensive staff involvement for individuals with high support needs moving from a congregate setting: *‘Its not just because of their disability, but because they have had a deprivation of opportunity and don’t know how community works. Now community has its own set of rules, doesn’t it?’* (ID27, telephone interviewee).
Staff lacking skills and inadequate staffing levels	1. Staff lacking skill as a local connector 2. Low staff levels and staff availability challenges 3. Staff challenged by service changes and lack of roster flexibility 4. Management of risk and ensuring personal safety	‘So, there is a difference between somebody accessing community facilities and actually participating in the community. And its that relationship that staff build with the people they are supporting and the people they are interacting with and them knowing when to step back and let things happen. Its building the capacity of other people. So, its that knowledge of, if I am right next to this person all the time, guiding and supervising and doing all of that, then this isn’t going to happen.’ (ID29, telephone interviewee).
Managing changes to new locally based models	1. Securing funding 2. Finding suitable accessible properties 3. Inadequate resources for new service models 4. Family fears and managing expectations for staffing/supervision 5. Insurance as a barrier 6. Lack of transport 7. Serving people in remote rural locations	*‘I suppose one of our difficulties there, is that largely, there is quite a lot of small town and rural that we operate within. So, for example, we have a local service in (name of town 1), but effectively identify that we serve four communities, the communities of (Names of four localities – towns and townlands) but our service is pretty much in (name of town 1). So, one of our difficulties is that being involved in community and trying to put a focus on as much as possible on being involved in the local community, but sometimes that is not as easy.’* (ID 08, telephone interviewee)

The first category of challenges included *concerns about the preconceptions and negative attitudes of local people*. This related to the challenges of building relationships and finding natural supports, particularly when moving into a new area. The issue of public acceptance was noted, with concerned groups of neighbours sometimes voicing objections before adults with intellectual disabilities moved into their new group home. Another service leader interviewed pointed to the difficulty of individuals with intellectual disabilities building relationships with other employees in work environments (see Table 4, ID08 quote).

The second category of challenges related to *individual characteristics of adults with intellectual disabilities with the potential to impact on engagement.* Individual factors such as lack of experience of living in a locality, fear of new experiences and acquiring social, communication, practical and decision-making skills, were reported to have the potential to impact engagement (see Table 4, ID27 quote). Individuals with challenging health care needs joining mainstream clubs may be daunting for other group members and their poor health may affect attendance. Poor motivation and commitment may lead to inconsistent attendance for others, thus impacting on forming connections. The specific challenges experienced by adults with behaviours of concern when finding suitable leisure groups/activities were noted, as were those faced by some adults with both intellectual disability and autistic spectrum disorder. Service providers highlighted the complexity of protecting the dignity of individuals with behaviours of concern, while balancing the needs of immediate neighbours and the potential negative effects on other group home residents, if neighbours reacted unfavourably. However, the commitment of service providers to the social inclusion in localities of adults with high support needs was underscored.

A third category of challenges centred on *inadequate staffing levels and staff lacking skills to facilitate social inclusion in localities.* Staff flexibility was reported as a concern when daytime hours were available, but evening hours were needed to support activities. Inadequate availability to support participation was reported to negatively affect engagement of individuals. Staff managing risk and ensuring personal safety were challenges also included in this category. Some staff were reported to be challenged by new service requirements and lacked skills in sourcing new opportunities or fostering relationships to develop. The sophisticated skills required by staff to build the capacity of people without disabilities to engage with adults with intellectual disabilities were highlighted, as illustrated by the quote from ID29 in Table 4.

The final category of challenges centred on *changing to new locally based service models.* Adjusting to the changes associated with new service models was reported to lead to challenges for some family members, including reducing staff ratios and concerns about safety. Finding suitable accessible buildings in the desired locations was a problem. Securing funding and the level of staff resources required for new locally based services for people with a wide variety of needs was reported as difficult. Transport was named as a general problem, most especially in rural areas. One service leader flagged the complexity of serving people living in a region with a range of localities (see quote ID08, Table 3). The impact of location on service delivery featured, with particular challenges when serving people living in a range of locations, including remote rural areas or impoverished localities, with few activities.

### Role of frontline staff in supporting social inclusion

All survey participants were asked to rate the importance of the role of frontline staff teams, including team leaders, in supporting social inclusion in neighbourhoods for adults with intellectual disabilities. Almost all (97.5%, n=39) rated it as either extremely important (80%, n=32), very important (15%, n=6) or important (2.5%, n=1). Content analysis of responses to a survey question on how frontline staff teams working in all service types best support adults with intellectual disabilities, resulted in four clusters (see Table 5).

**Table 5.** Roles of Staff and Families in Supporting Social Inclusion in Neighbourhoods.

Clusters related to role of staff	Sample quotes
The role of staff as connectors/bridges	*‘Staff facilitate networking and developing inclusive activities and opportunities. From the neighbourhood point of view, they are available as resources to help dispel myths about a person with disabilities that they may have.’* (ID22, survey respondent)
Staff attitudes to social inclusion in neighbourhoods.	*‘The attitude and approach taken by staff members can impact upon the interactions the individual they are supporting has with others when in the community and it is important that these interactions are positive.’* (ID29 survey response) … *‘We would have some long-standing staff who are very caring, supportive of people, and there is a level where it is too supportive.’* (ID29, telephone interview response)
Specific staff skills in fostering social inclusion in neighbourhoods	*‘Staff must know what pace they should work at with the service user. Staff must ensure that the circle of support (family, [specific job title], staff, key worker, manager) are all supporting the same plan.’* (ID39, survey response).
	*‘So when you are working with people like that [*an adult with intellectual disability and autistic spectrum disorder, with behaviours of concern occurring in public places*] and when you know that there are challenges, the big thing is, when you are making a plan about engaging the local community and your local facilities, is your exit plan. If it goes pear shaped, how are we getting out of this?’* (ID08, telephone interviewee)
The role of frontline managers and multi-disciplinary teams	*‘The team leader/manager: listening to staff; discussing challenges; teaching staff to be companions and what that means, doing with’* (ID30, survey response).
Clusters related to role of families	Sample quotes
Families as natural support networks	*‘Adults with intellectual disabilities need to have the option to make decisions about their own lives which are not in agreement with the families.’* (ID11, survey response)
Role of families as bridges/connectors	*‘Family members are generally well known within the community and, as such, can be a useful conduit for the individual. However, their level of support and which family member provides that support, has to be at the discretion/request of the individual.’* (ID28, survey response)
	*‘I think when we talk about participation, we would get lots of well-meaning stuff around supporting people [from families]. It doesn’t translate into, there is only a percentage of that that translates into reality’* (ID08, telephone interviewee)
Family members as role models, teachers of new skills and/or advocates	‘…*“families who rate community participation high in their own lives can by example support adults with intellectual disabilities to participate too”’* (ID05, survey response).
	*‘Families are usually the fiercest advocates, and this should be used. The family are a huge resource of information and history. The family usually already has community connections that could be accessed. Families can begin the promotion of valued roles within the home or family unit and provide grounds to be built upon. Often families need to go through a difficult change themselves and if they embrace it the benefits for their family members [with intellectual disability] can be immense.’* (ID40, survey response)
The importance of adult siblings and extended family	*‘Family supports are often entangled with the dilemma between positive risk taking and capacity building for their family member and wanting to be assured they are safe. Siblings have a huge role to play in helping set goals and expectations that might otherwise be “safer”’.* (ID02, survey response)
	*‘Some families support residents very well and siblings help residents access community. Other families see this as staff job.’* (ID28, survey response).

First, participants highlighted *the role of staff as connectors/bridges* for adults with intellectual disabilities into localities. A facilitation role was evident, with staff opening up inclusive opportunities or activities and addressing barriers to accessibility. This included staff through their own personal social networks supporting individuals to participate and to make connections. It also included an intentional fostering of relationships with neighbours, facilitating positive introductions and helping with communication challenges. A second cluster of responses focused more specifically on *staff attitudes to social inclusion in neighbourhoods*. Staff members’ attitudes to social inclusion in neighbourhoods were reported to vary and to have the potential for positive and sometimes unintended negative consequences on adults with intellectual disabilities making connections. A shift in emphasis from staff ‘doing for’ to empowering individuals was noted, as illustrated in the quote by ID29 in Table 5 about long-standing staff who may be ‘too supportive’. In addition to attitudes, *specific staff skills in fostering social inclusion in neighbourhoods* were identifiable as a cluster, including developing a holistic knowledge of peoples’ interests and support needs, which may be based on circles of support or other individualised planning models (see ID39 quote, Table 5). Respondents highlighted skills in sourcing information in a locality, preparing well, teaching a range of new skills and building confidence. The specific skills in introducing people who communicate without using language to strangers or supporting individuals with behaviours of concern to engage in their locality were also noted, with an illustrative example from a service leader (see ID08 quote, Table 4). Finally, the ability of staff to fade out and mobilise natural local supports where possible, so that connections are sustained in the long-term, was also a valued skill.

The final cluster highlighted *the role of frontline managers and multi-disciplinary teams*, with robust systems and processes to manage risks, teach staff necessary skills, structure reviews and maintain clear communications, (as identified in the quote by ID30, see Table 4). Continued involvement of a multi-disciplinary team was also seen as valuable, in particular in supporting transitions from institutional care to a neighbourhood location context.

### Role of families in supporting social inclusion

All survey participants were asked to rate the importance of the role of families in supporting social inclusion in neighbourhoods of adults with intellectual disabilities. Most (90%, n=36) rated it as important, either extremely important (47.5%, n=19), very important (35%, n=14) or important (7.5%, n=3). Four clusters of responses were identified (see Table 5).

In the first cluster, *families were represented as natural support networks* and were acknowledged as having the potential to offer benefits for their relative with intellectual disability engaging in their locality. This cluster included reference to key changes and transitions in an adult’s life (e.g. moving to a new locality) that were reported as presenting concerns for families. Other challenges included those faced by elderly parents and rural isolation for individuals with intellectual disabilities, depending on the location of the family home. Some service leaders perceived a tension between an adult’s growing independence, choice and decision-making and family concerns about risk and safety (see quote by ID11, Table 5). The natural *role of families as bridges/connectors* formed a second cluster of responses, with an emphasis on utilising their local knowledge and existing connections to open up opportunities for their relative. Family involvement in individual planning processes and their engagement by service providers as a resource with local knowledge was highlighted. Their unique position was underscored, with family members often already embedded in the locality in which their relative lived (or grew up in) and in which opportunities to engage were available. However, the challenge was highlighted of welcoming the natural support offered by families, while respecting the autonomy of an adult with intellectual disability regarding the level of their engagement, (as illustrated in the quote by ID28, Table 5). In contrast, other service leaders reported practice experiences that led to some doubts about whether family members’ intentions translated into actions that supported their relatives' local engagement (as illustrated in the quote by ID08, Table 5). *Family members as role models, teachers of new skills and/or advocates* formed a third cluster of responses, with an understanding that knowledge and skills to engage in a locality needed to be learned (see ID05 quote, Table 5). Participants also highlighted the role of families as advocates, alongside the ‘difficult change’ they may need to make to embrace social inclusion, as illustrated in the quote by ID40, Table 5. In the final cluster, *the importance of adult siblings and extended family* was identified by service leaders as creating opportunities for adults with intellectual disabilities to engage in a locality, with invitations to extended family gatherings/events mentioned. The value of adult sibling support was noted (see quote by ID02, Table 5), though mixed perceptions were reported among family members about whether they or staff were responsible for supporting social inclusion (as identified by ID28, Table 4). One service leader acknowledged that some adults had two localities that they could engage with and saw a role for the extended family in supporting individuals to retain connections in the locality they grew up in, if they had moved out of their family home.

### Mainstream organisations and professionals supporting social inclusion

All bar one of the service leaders confirmed that their organisation had linked with mainstream groups or local people, with a view to fostering the inclusion of adults with intellectual disabilities they served. A diverse range of local clubs or groups (n=187) were listed, including those dedicated to sports/health/leisure or faith-based groups and local businesses/charities offering employment or volunteering opportunities. In addition, 127 mainstream funded organisations were listed by many survey participants (62.50%, n=25) who confirmed that their service had also forged these links to further social inclusion opportunities. A range of mainstream organisations were listed, from regional community development and education bodies to local community centres and health/social services. Service providers seeking to partner with employers was also evident through regional business development networks or regional community employment or volunteering schemes. National/provincial funding bodies were also listed, indicating that some service providers may have sought additional funding for projects to promote social inclusion in localities. A full breakdown of all organisation types, whether locally based voluntary groups or mainstream funded organisations is available in Supplemental Material 2.

## Discussion

This study examined service providers’ actions or interventions, if any, to support adults with intellectual disabilities to participate in their neighbourhoods**.** Almost all reported that they offered service initiatives supporting social inclusion in localities. Shifting towards new service models (including outreach services) was most often mentioned as boosting social inclusion. Services based in localities were combined with direct support staff adopting the proactive role of community connector. Understanding service providers and staff as part of the scaffolding that supports social inclusion in neighbourhoods, confirmed prior review findings (Howarth et al., 2016), including people with behaviour labelled as challenging (Bigby, 2012). While person-centred planning was indicated in some actions supporting individuals, an in-depth examination of person-centred planning was outside the scope of the present study. Where and with whom an adult with intellectual disability lives was identified as underpinning opportunities for social inclusion in neighbourhoods, confirming prior empirical findings that segregated residences for groups runs counter to social inclusion in neighbourhoods for individuals (Baker, 2007; McCausland et al., 2016; McConkey et al., 2005, 2007). In addition, the present study also found that new models of day services following objectives contained in the relatively recent ‘New Directions’ (HSE, 2016) reform of day services policy had begun to take root. Local hubs and outreach services were described, with a shift away from large groups of adults being served during daytime hours in service buildings that were at a remove from local amenities. A study by Power (2013), including government officials and service provider managers (among other stakeholder groups) in both Ireland and Canada, found that the process of meaningful social inclusion was likely to take time, requiring strong leadership and the building of a wide inventory of local options. Results of the present study indicated that some Irish service providers had accessed a wide-ranging list of local clubs and organisations that individuals had been supported to join. Participants also listed mainstream funded bodies that had been approached by service providers, with the intention of forming strategic partnerships. Some service leaders recommended offering their disability expertise to mainstream local planning processes supporting social inclusion, thus moving beyond the boundary of the specialist intellectual disability service organisation. This represents an addition to the role of service providers as solely lobbying for scarce resources for specialist intellectual disability services. Shifting to new service models was recognised by some as requiring direct support staff recruitment, education and deployment planning. This finding reflects in part the examination by Thorn et al. (2009) of an intervention to change the systems and culture of a large residential facility transitioning to locally based services, in which an investment in staff education was a key component. It was unknown at the outset of the present study that some Irish service providers had created dedicated staff roles intended to support social inclusion on an organisation-wide basis. This is a new development in the literature on this topic and the effectiveness of such specialist staff roles merits further enquiry.

This study captured an array of service initiative examples that were deemed by service leaders as broadly helpful in achieving social inclusion outcomes. Social roles as focal points for social inclusion in neighbourhoods featured in practice examples summarised by service leaders, linked to goal setting in individualised planning processes (e.g. McConkey and Collins, 2010). This is consistent with prior studies in which valued social roles were stepping stones to engagement (e.g. Lemay, 2006). However, being acquainted with fellow employees did not appear to extend to contact outside the workplace. Underpinning the success of social roles was the importance of common interests, with examples of volunteering with a local tidy up group or shared leisure interests such as membership of a walking club. This confirms findings on the value of common interests (e.g. dog walking) as opportunities for convivial encounters or an integrating shared activity as a focal point for successful engagement with non-disabled peers (e.g. Bould et al., 2018; Wilson et al., 2010). The positive outcomes for Irish localities reported by participants, offered tangible examples of adults with intellectual disabilities contributing (O’Brien and Mount, 2015) and being recognised publicly as competent and valued (Cobigo et al., 2012). However, these reported outcomes are solely from the standpoint of service leaders. Participants identified both successes and challenges to social inclusion. However, the experiences of other stakeholders, in particular people with intellectual disabilities, on the effectiveness of support from service provider staff to boost their engagement locally may yield different findings.

Service leaders clearly outlined challenges linked to individual characteristics, public acceptance as well as practical aspects such as available transport or accessibility of buildings and mainstream activities to suit adults with intellectual disabilities. The complex challenge for staff was evident in the need to protect the dignity of individuals with behaviours of concern or complex medical needs to engage in their locality, while balancing the needs or concerns of neighbours. Staff members providing individualised support was recognised as scaffolding for social inclusion in a locality. However, staff lacking skills or motivation in the role of local connector, inflexible work practices or inadequate staffing levels while potential barriers, are not new challenges, having been reported previously (e.g. Bigby and Wiesel, 2015; Zakrajsek et al., 2014). For Irish service providers serving broad geographic areas, with single service hubs serving a range of local areas, the question of which locality to invest staff resources in to build neighbourhood connections for individuals, is a complex challenge that merits further enquiry.

The natural support role of family members as connectors in a locality, while valued by service providers for their local knowledge and networks, was counterbalanced by challenges regarding an adult’s decision-making autonomy alongside family concerns about risks. Prior literature identifies an actively supportive family who understands participation as an enabling factor (e.g. McConkey and Collins, 2010; Walker et al., 2014). In the present study, Irish service providers reported family involvement as channelled in the main through individualised planning processes and some recognised family members as role models, teachers and advocates for their relative with intellectual disability. Some considered adult siblings and extended family members as positive contributors. However, while almost all survey respondents rated the role of family members as important, no service-wide initiatives were mentioned by those surveyed that focused on groups of family members being facilitated to open up opportunities for social inclusion in neighbourhoods. In addition, the finding that some adults with intellectual disabilities are connected to two localities, (e.g. living in a group home mid-week and in the neighbourhood of the family home at weekends), is not mentioned in the prior literature and merits further exploration.

### Strengths and limitations

The choice of mixed method survey with the addition of follow-up interviews was effective. Testing the survey content, the advance information materials, and the operational procedures for launching the survey lent authenticity to the study. Content analysis was applied with consistency and credibility checks added to the trustworthiness of the process. The mixed methods approach also had the advantage of offering a rich integration of findings from both the survey and the interviews. A key strength was reaching a large geographically-dispersed national sample using a cost-effective online survey. This survey of service providers for adults with intellectual disabilities attracted a reasonable response rate. Interest in the intellectual disability sector on the topic of social inclusion in neighbourhoods may be inferred from the high rate of full survey completion and the proportion of respondents who consented to be included in the selection pool for interview. However, the perspectives of service leaders alone are represented in the findings of this study. Further research on the efficacy of service initiatives on social inclusion from the perspective of adults with disabilities themselves is recommended. The Irish intellectual disability service context is complex as reflected in service provider size, funding category and geographic localities served. A strength of the study was that the sample reflected the range of service provider types, including the more recently developed private sector. Although clear categories of leadership roles were discernible, respondents’ job titles varied considerably, raising a potential question about the homogeneity of the sample. However, a strength was that the CEOs/service leaders responding were long-standing, highly-experienced staff. On balance, this sample can be taken to be representative of the range of service providers for adults with intellectual disabilities in the Republic of Ireland, lending ecological validity to the enquiry.

### Practice and research implications

While almost all Irish service providers gave indicative examples of supporting social inclusion in localities, none reported that these initiatives had been independently evaluated. Developing reflective process evaluations (e.g. Guerin et al., 2017) of social inclusion projects and systems is proposed as a possible way forward. It is recommended that these evaluation studies would include all stakeholders to establish the factors leading to successful outcomes, challenges to be addressed and to identify unintended service obstacles to progress.

Person-centred planning was referenced in some of the examples offered. An intervention study is recommended that tests the effectiveness of person-centred planning on outcomes for social inclusion in neighbourhoods, given prior review findings indicating that this framework has positive outcomes for what authors describe as community involvement of adults with intellectual disability (Ratti et al., 2016). Given the lack of practice-based group interventions for family members, it is recommended that this proposed study may be combined with a sample of adult siblings nominated by their brother/sister with intellectual disability to participate in supporting the achievement of social inclusion goals, using their local knowledge and their social network. The study may also examine the tension as expressed by some service providers between the decision-making autonomy of adults with intellectual disabilities and the involvement of their family members in supporting social inclusion. Irish service providers were challenged to identify unpaid people in mainstream settings to support social inclusion of their neighbours with intellectual disabilities. A replication study that tests the recruiting of mentors in mainstream local clubs and organisations in the Irish context, may be a potential bridge to addressing this practice challenge. With regard to promising interventions, a cluster of Australian studies have reported the testing of an intervention in which volunteer club mentors supported adults with intellectual disabilities to join leisure clubs on an equal footing. These papers offer a clear conceptual framework and best practice recommendations to service providers on supporting social inclusion (Bigby et al., 2014; Wilson et al., 2010, 2013). Bigby et al. (2018) found that these and other related studies provided promising approaches which had the potential for larger scale replication and examination of outcomes. Replication across countries and cultural contexts is recommended, with the findings of the present study suggesting that individuals are mentored to acquire valued social roles in which a visible/tangible contribution to their locality is the goal. Related to the lack of local people to offer support, the present study indicated that being employed did not generally lead to leisure or social engagement with colleagues outside the workplace. However, the workplace factors underpinning this have not been comprehensively examined and this too merits further research exploring multiple perspectives.

Barriers such as the gaps in knowledge that an individual with intellectual disability may have about what is happening in their neighbourhood featured in this study. Developing an education module arising from the findings of the present study is recommended. Developing the content and piloting of this module as an intervention involving a sample of stakeholders is recommended, including participants with intellectual disabilities, direct support staff, frontline managers, clinical staff and family members.

A practice challenge to overcome identified by providers serving broad geographic areas with multiple neighbourhoods, is the question of which locality to invest staff resources in to build local connections for individuals. This is a complex challenge which merits further enquiry. Unknown at the outset of the present study was that some Irish service providers had created dedicated social inclusion staff roles which carried cross-organisational responsibility for opening up social inclusion opportunities. The survey did not allow for the examination of these roles and such specialist roles are not examined in the prior literature. A recommendation for further research is an enquiry into the scope of these roles and the practice knowledge of the post holders as local connectors, across a range of neighbourhood and locality types.

Choice of geographic location in which to purchase or rent individualised or small group accommodation for adults with intellectual disability is a key contributor to offering rich opportunities for neighbourhood participation. Safe localities are recommended with a range of services, social and leisure activities, a range of social role and volunteering opportunities, and accessible public transport links (and/or most amenities within walking distance). There is scope to develop a comprehensive neighbourhood/wider locality assessment tool which may be useful prior to purchasing or renting accommodation for either individuals with intellectual disabilities living alone or in small group homes, in addition to day service premises serving larger groups. It is recommended to develop this tool with the specialist social inclusion post holders identified above. Further examination of the impact of geographic location (urban, suburban, town and rural) on the opportunities for neighbourhood engagement for adults with intellectual disabilities is proposed from the perspective of these specialist social inclusion staff.

## Conclusion

To the authors’ knowledge, this is the first study with a national service leader sample spanning all intellectual disability service provider types, that specifically explored practice initiatives in supporting social inclusion in neighbourhoods. Service providers play an important role in providing individualised supports that foster local engagement for adults. However, the Irish intellectual disability service context is complex and service leaders have reported many challenges that may impede progress on fostering local engagement. The findings of this study capture the changing roles of staff adjusting to new service models and reveal an enhanced role for families in supporting the social inclusion in their neighbourhoods of adults with intellectual disabilities. Results also point to service strategies at two levels, including the micro level of supporting individuals to achieve their social inclusion goals. Operating outside of traditional organisational boundaries, macro-organisation-wide initiatives also serve adults with intellectual disabilities, with the potential for service providers to create strategic partnerships with mainstream bodies already promoting social inclusion. The findings of this study highlight some of the practice challenges of implementing the social inclusion aspects of the UNCRPD (2006). While this study was located in Ireland, its findings as a national study of service providers may be considered relevant to other countries in transition from serving adults with intellectual disabilities in congregate or campus settings to locally based services intended to foster social inclusion.

## Supplemental Material

Supplemental Material - Supporting Social Inclusion in Neighbourhoods of Adults with Intellectual Disabilities: Service Providers’ Practice Experiences Click here for additional data file. Supplemental Material for Supporting Social Inclusion in Neighbourhoods of Adults with Intellectual Disabilities: Service Providers’ Practice Experiences by Geraldine Boland and Suzanne Guerin in Journal of Intellectual Disabilities

Supplemental Material - Supporting Social Inclusion in Neighbourhoods of Adults with Intellectual Disabilities: Service Providers’ Practice Experiences Click here for additional data file. Supplemental Material for Supporting Social Inclusion in Neighbourhoods of Adults with Intellectual Disabilities: Service Providers’ Practice Experiences by Geraldine Boland and Suzanne Guerin in Journal of Intellectual Disabilities

Supplemental Material - Supporting Social Inclusion in Neighbourhoods of Adults with Intellectual Disabilities: Service Providers’ Practice Experiences Click here for additional data file. Supplemental Material for Supporting Social Inclusion in Neighbourhoods of Adults with Intellectual Disabilities: Service Providers’ Practice Experiences by Geraldine Boland and Suzanne Guerin in Journal of Intellectual Disabilities

Supplemental Material - Supporting Social Inclusion in Neighbourhoods of Adults with Intellectual Disabilities: Service Providers’ Practice Experiences Click here for additional data file. Supplemental Material for Supporting Social Inclusion in Neighbourhoods of Adults with Intellectual Disabilities: Service Providers’ Practice Experiences by Geraldine Boland and Suzanne Guerin in Journal of Intellectual Disabilities
