# Supplementary material for: Supporting Social Inclusion in Neighbourhoods of Adults with Intellectual Disabilities: Service Providers’ Practice Experiences
Source: J Intellect Disabil. 2022 Apr 21;27(2):291–314. doi: 10.1177/17446295221085479 (PMC10164231; doi:10.1177/17446295221085479)
Supplement: Supplemental Material - Supporting Social Inclusion in Neighbourhoods of Adults with Intellectual Disabilities: Service Providers’ Practice Experiences [file sj-pdf-1-jld-10.1177_17446295221085479.pdf]

Supplemental Material 1

Online Survey

[Logo of academic institution blinded for peer review]

Launch page of survey.

A National Survey of Irish CEO's / Directors of Intellectual Disability Services.

Introduction.

Thank you for consenting to complete this national online survey of CEO's/ Directors of Services in organisations serving adults with intellectual disabilities. This study aims to survey service providers of supports for adults with intellectual disabilities in Ireland. The core objective is to explore awareness of social inclusion as it impacts on neighbourhood connections of adults with intellectual disabilities. The survey also considers the resources/ supports provided, as identified by CEO's and/or Directors of Services. These may include policies, actions, or interventions (if any), to support adults with intellectual disabilities to participate in their neighbourhoods.

The central research question of the proposed survey is:
How are adults with intellectual disabilities living in dispersed housing in Ireland best supported to participate satisfactorily in their neighbourhoods?
All information that you offer as part of the survey will remain confidential (see CEO/ Director of Services information sheet for details). Please read each question carefully. Some questions are factual, others will ask you to draw examples from your management experiences of the current service that you lead.
Some questions ask about your organisation's policies as they relate to neighbourhood participation. You might find it helpful to have these to hand before you start completing the survey.
The survey takes about 30 minutes to complete. It is best completed without interruption on a desktop computer or laptop, in one sitting.

Question 1.
First, considering the area of policy-
Is there any organisational policy on neighbourhood participation for adults with disability in the service that you lead?

Please tick (✓):

- ◊ Yes
- ◊ No
- ◊ Not sure

(Note: With skip logic, respondents who replied No or Not Sure proceeded directly to Question 2.)

Question 1a
Please type in the exact title of the policy in your service that relates to neighbourhood participation for adults with intellectual disabilities, in the box below.

Question 1b
In your own words, write 3 to 4 sentences that summarises the aims of this policy, specifically as it relates to the neighbourhood participation of adults with intellectual disabilities.

Question 2.

Next, based on your experience in services for adults with intellectual disabilities, you are asked to think about how neighbourhood participation might be defined.

Considering the adults with intellectual disabilities being supported in services that you lead; how would you best define what the concept of neighbourhood participation means?

Please write 3 to 4 sentences on how you define neighbourhood participation, as you understand it for adults with intellectual disabilities who wish to participate.

Question 3.
Which of the following aspects do you think should be included in a definition of neighbourhood participation for adults with intellectual disabilities?
Please tick any of the options below. Feel free to tick as many or as little as you think belong in a definition of neighbourhood participation for adults with intellectual disabilities.

- ☐ Being known to see or by your name in a locality
- ☐ Doing some of your shopping in your neighbourhood
- ☐ Accessing some health services locally (e.g., G.P., dentist)
- ☐ Having the choice to engage in voluntary work
- ☐ Having family or friends living in the locality that you see as often as you want
- ☐ Having a sense of connection to the place
- ☐ Having a sense of belonging with people who live locally
- ☐ Engaging in leisure activities in the locality
- ☐ Being a member of local organisations (e.g., church group, club member)
- ☐ Knowing what's happening in the area

Question 4.

Moving on to specific actions or projects that services might undertake to support participation.
Has your organisation been involved in any initiatives specifically aimed at supporting an adult or adults with intellectual disability to participate to their level of satisfaction in their neighbourhood?
Please tick (✓):
Yes No Not sure
(Note: With skip logic, respondents who replied Yes proceeded to questions 5 and 6, those who replied No or Not Sure proceeded directly to Question 7)

Question 5.
Learning from practice is important to this research on neighbourhood participation.
Please describe in detail an initiative that your organisation was or is involved in, aimed at supporting an adult or adults with intellectual disabilities to participate in their neighbourhood.
In the separate boxes below, please give full details of:
1) the specific objective of the support action;
2) a summary description;
3) who was involved;
4) the challenges faced; and
5) what the outcome was.
Please feel free to include an initiative that had a desired result or one that was not so successful.

1) The specific objective of the support action

2) Describe the project or the action briefly

3) Who was involved? (Please list)

4) Any challenges faced? (Please outline in detail)

5) What was the outcome? (Please outline in detail)

Question 6.
How satisfied are you with the outcome of the action or initiative you described to support neighbourhood participation?

- o Very dissatisfied
- o Dissatisfied
- o Neither satisfied nor dissatisfied
- o Satisfied
- o Very satisfied

Question 7.
Next, this survey asks you to consider the role of staff in supporting neighbourhood participation.
In what ways do you think that adults with intellectual disabilities are best supported by frontline staff teams (including the frontline manager) in all service types to participate in their immediate neighbourhoods?
Please write 4 to 5 sentences.

Question 8.
How do your rate the role of frontline staff teams (including frontline managers) in supporting neighbourhood participation of adults with intellectual disabilities?

- Not important
- Somewhat important
- Important
- Very important
- Extremely important

Question 9.
How do think that service providers at an organisational/strategic level can best support adults with intellectual disabilities to participate in their neighbourhoods?
Please write 4 to 5 sentences in the space below

Question 10
How do your rate the role of service providers at an organisational/strategic level in supporting the neighbourhood participation of adults with intellectual disabilities?

- Not important
- Somewhat important
- Important
- Very important
- Extremely important

Question 11
Next, we will consider the role of family members (including both immediate and extended) in supporting neighbourhood participation.
In what ways do you think that adults with intellectual disabilities are best supported by family members to participate in their neighbourhoods?

Please write 4 to 5 sentences

Question 12.
How do your rate the role of family members (including immediate and extended) in supporting the neighbourhood participation of adults with intellectual disabilities?

- Not important
- Somewhat important
- Important
- Very important
- Extremely important

Question 13.
In some localities in Ireland, there are informal structures: clubs (e.g., mens' sheds); local groups; or people (local champions/ volunteers) who may facilitate adults with ID to participate in their neighbourhood.
Has your service had an opportunity to link with any of these clubs, groups or local people to support adults with ID to participate in their neighbourhoods?
Yes No.
(Note: With skip logic, respondents who replied Yes proceeded directly to Question 14, those who responded No were directed to question 15.)

Question 14.
Please list examples of the clubs, local groups (title only) and /or name the volunteer role of local champions/ community connectors that your ID service has linked with, with the goal of facilitating adults with ID to participate more in their neighbourhoods.

1.
2.
3.
4.
5.
6.
7.

Question 15.
In some localities in Ireland, there are **formal funded structures** (e.g., a local county council) **or professionals** (e.g., an interested community development worker) that may facilitate adults with ID to participate in their neighbourhood.

Has your service had an opportunity to link with any funded community organisations or professionals (outside of ID services) to support individuals to participate in their neighbourhoods?
Yes No.
(Note: With skip logic, respondents who replied Yes proceeded directly to question 16, those who responded No were directed to question 17.)

Question 16.
Please list the formal community structures (titles only) or name the job titles of local professionals (non-ID related) that your organisation has linked with the goal of facilitating adults with ID to participate more in their neighbourhood.

1.
2.
3.
4.
5.
6.
7.

Question 17.

This section asks you some questions about your organisation.
First, you are asked about the type of organisation, based on how it is funded.

Please tick (✓) the relevant box below:

The service I lead is:

- A section 38 (Health Act, 2004) voluntary organisation
- A section 39 (Health Act, 2004) voluntary organisation
- A HSE directly managed service
- A 100% privately funded company
- Other (Please specify the funding arrangement in the box below)

Question 18.

Please tick (✓) the relevant box below:

Please indicate the size of your organisation, in terms of the number of adults with intellectual disabilities living in non- campus settings or living with family members, served in all service types.

- ◊ Fewer than 200 adults with ID served
- ◊ Between 200 and 1500 adults with ID served
- ◊ Over 1500 adults with ID served

Question 19.

Which type of geographic location do adults with ID served by your organisation live in? Please tick the type of locations from the listing below:
(Note: if your organisation serves adults in a mix of geographic locations, please tick all that apply)

- ◊ Urban/ suburban
- ◊ Town
- ◊ Rural village
- ◊ Remote rural

Question 20.

The next section asks you a little about yourself.

Please type your exact job title in the box provided below:

Question 21.

Please tick (✓) the relevant box below:

I have been employed in my current role as CEO / Director of Services (or alternative title) for:

- Less than 12 months
- 13 months – 24 months
- Between 2 years -5 years
- Between 5 years- 10 years
- Between 10 years -15 years

- Between 15 years-20 years
- 20 plus years
- Prefer not to answer

Question 22.
Please specify in the box below, the total number of years that you have worked with your current organisation in the format: years and months (e.g., 10 years and 7 months).
Example
Years

**10**

Months

**7**

Question 23.
Do you have any final comments on how your service organisation supports the neighbourhood participation of adults with intellectual disabilities, that were not covered in previous questions?
Yes **No**
(If Yes is selected, skip logic will display question 24. If No is selected, respondents will be prompted to proceed to question 25)

Question 24.
Please write any final comments on how your service organisation supports the neighbourhood participation of adults with intellectual disabilities in the box below.

Question 25.
When this online survey is closed, I intend to complete a small number of follow up telephone interviews with a sample of participants during the first half of 2019. The aim is to develop a more comprehensive understanding of the findings from the survey.
Would you like your name to be included on a list of respondents to the survey who may be invited to participate?
Yes No
(Please note that ticking Yes does not commit you to being interviewed, only to having your name on a list of potential interviewees. You may decline at a later stage.)
(If Yes is selected, skip logic will display Question 26. If No is selected, respondents are directed to the closing page of the survey).

Question 26.
Thank you for agreeing to me adding your name to a list of survey respondents who may be invited to complete a follow up telephone interview. Please type your email in the box below.
(Reminder: offering your email does not commit you to being interviewed, only to having your name on a list of potential interviewees. If selected for interview, you may decline at a later stage.)

Closing page of survey.

Thank you for completing this survey. Your time and your contribution to research for people with intellectual disabilities in very much appreciated.
