# Supplementary material for: Supporting Social Inclusion in Neighbourhoods of Adults with Intellectual Disabilities: Service Providers’ Practice Experiences
Source: J Intellect Disabil. 2022 Apr 21;27(2):291–314. doi: 10.1177/17446295221085479 (PMC10164231; doi:10.1177/17446295221085479)
Supplement: Supplemental Material - Supporting Social Inclusion in Neighbourhoods of Adults with Intellectual Disabilities: Service Providers’ Practice Experiences [file sj-pdf-2-jld-10.1177_17446295221085479.pdf]

Supplemental Material 2

Adults with intellectual disability participating in their neighbourhoods.

A national survey of Irish service providers.

Phase 2- Telephone interviews with a sample of survey respondents

Telephone interview topic guide

Objective: To reflect key findings of the survey back to a sample of participants who completed online surveys, seeking more in-depth comments, with an emphasis on exploring the practice experiences in organisations.

Topic 1: Role of participant within organisation and demographics.

Probe: Can you tell me a little about your role within the organisation?

Probe: I need to capture some demographics of the sample of people completing interviews. Can I check with you if you are comfortable telling me your age range (10 year ranges prompted) and preferred gender identity?

Topic 2. Service initiatives supporting adults with intellectual disabilities in their neighbourhoods.

Preamble: Learning from practice in organisations is important to this research. The survey has given me a good sense of the type of work that your organisation is doing, in supporting adults to participate in their neighbourhoods and I am keen to explore your views on what has supported or inhibited that work.

Probes:

Do you have any specific examples from initiatives planned by your organisation that show us what works best in supporting adults to participate in their neighbourhoods?

And what would say are the main obstacles that your service has met with in planning initiatives that support adults to participate in their locality?

Do you have any specific examples from practice that show us the main obstacles that your organisation has encountered?

Topic 3. Role of staff in supporting neighbourhood participation of adults with intellectual disability

Probes:

The literature on community participation tells us that staff are crucial supports for neighbourhood participation. In your organisation, are there any examples that stand out in which the role of staff to facilitate adults to engage with the people and places in their neighbourhood was important to a positive outcome?

The literature also tells us that sometimes staff practices may block adults engaging and forming connections in their neighbourhood. Are there any staff practices that you are aware of in your service that perhaps, without intending to, block adults engaging with and forming connections in their neighbourhood?

Topic 4. Role of family members (including both immediate and extended) in supporting neighbourhood participation.

Probe: Can you offer any specific examples of structured initiatives in your service in which the family members of adults have been engaged with in a planned way, with the objective of supporting their relative to participate more in their neighbourhood?

Topic 5. Support people and organisations in localities that facilitate adults with ID to participate.

Probe: The survey findings indicate the existence in some Irish localities of natural champions, people outside of services who can facilitate adults with ID to participate in their localities. Has your organisation had an opportunity to engage with this type of community leader?

Similarly, there are clubs or organisations in localities that are welcoming of members with intellectual disabilities. Has your organisation had an opportunity to engage with this type of club or organisation?

Topic 6. Role of organisational strategy in supporting neighbourhood participation.

Probe: Moving on now to the role of strategy and organisation wide planning focused on neighbourhood participation and belonging. Has your organisation had the opportunity to include neighbourhood participation for adults in either service or strategic plans?

Probe: What specific objectives related to of neighbourhood participation are included in your service or strategic plans?
