# Supplementary material for: Supporting Social Inclusion in Neighbourhoods of Adults with Intellectual Disabilities: Service Providers’ Practice Experiences
Source: J Intellect Disabil. 2022 Apr 21;27(2):291–314. doi: 10.1177/17446295221085479 (PMC10164231; doi:10.1177/17446295221085479)
Supplement: Supplemental Material - Supporting Social Inclusion in Neighbourhoods of Adults with Intellectual Disabilities: Service Providers’ Practice Experiences [file sj-pdf-3-jld-10.1177_17446295221085479.pdf]

Supplemental Material 3-Data analysis

Steps in Operationalising Inductive Content Analysis of Qualitative Survey Data

	Sequence of content analysis steps	Process of implementation for the qualitative survey data
Step 1	Reading all data repeatedly to achieve immersion and obtain a sense of the whole	Data extracts were read and re-read
Step 2	Coding of 'key thoughts or concepts' from the text data extracts	Codes were generated from the data extracts
Step 3	Sorting of codes into categories/ clusters. Refining and labelling of categories/ clusters	Codes were first sorted alphabetically. Grouping of codes into clusters was completed with provisional titles identified
Step 4	Clarifying links or relationships between categories/ clusters. (Map/ diagram)	Mapping of clusters/categories was completed, with diagrams
Step 5	Developing summary descriptors for each category/cluster.	Summary descriptors were developed
Step 6	Identifying exemplars for codes and categories/clusters from the data.	Illustrative examples of data extracts and codes for each category/cluster
Step 7	Reporting	Paper drafted reporting results

Note. Adapted from Mayring, P. (2000). Qualitative content analysis. *Forum: Qualitative Social Research, 1*(2) and Hsieh, H., & Shannon, S. E. (2005). Three approaches to qualitative content analysis. *Qualitative Health Research, 15*(9), 1277-1288. Copyright Sage journals.
