# Supplementary material for: Supporting Social Inclusion in Neighbourhoods of Adults with Intellectual Disabilities: Service Providers’ Practice Experiences
Source: J Intellect Disabil. 2022 Apr 21;27(2):291–314. doi: 10.1177/17446295221085479 (PMC10164231; doi:10.1177/17446295221085479)
Supplement: Supplemental Material - Supporting Social Inclusion in Neighbourhoods of Adults with Intellectual Disabilities: Service Providers’ Practice Experiences [file sj-pdf-4-jld-10.1177_17446295221085479.pdf]

Supplemental Material 4

Table 1

Local Groups, Clubs, Employers or Organisations (n=187) Engaged with by ID Service Providers

Category of club/ local group	N	%
Sports clubs	34	18.18
Gendered social clubs (e.g., men's sheds)	26	13.90
Craft/ art group or performance arts	22	11.76
Clean up/ tidy district groups	19	10.16
Faith based groups	15	8.02
Specialist leisure/sports club for people with disability	13	6.97
Local charity-funding/support/volunteering opportunity	11	5.88
Commercial healthy living group-weight loss or gym	8	4.28
Festivals or neighbourhood gatherings	5	2.67
Gardening/allotments	4	2.14
Local businesses (employment and work experience opportunities)	7	3.74
Retirement groups	6	3.21
Youth groups	3	1.60
Classes/education	2	1.07
Other	6	3.21

Note. Where clearly common titles or group types were identifiable, these have been clustered and aggregated data are presented.

Table 2

Mainstream Funded Structures or Organisations (n=127) Engaged with by ID Service Providers

Category of organisation	N	%
College (national/ regional)	27	21.26
Regional community development organisation/ network	18	14.17
Local authority	15	11.81
Mainstream community centre	12	9.45
Regional employment or volunteering organisation/ network	12	9.45
Local arts/cultural facility or library	8	6.30
National/provincial social inclusion or philanthropic funding organisation	8	6.30
National/provincial sports body	6	4.72
Local health/social services	5	3.94
National mainstream service organisation	4	3.15
Regional business development network	4	3.15
Rural transport company	2	1.57
Other	6	4.72

Note. Where common titles or group types were clearly identifiable, these have been clustered and aggregated data are presented.
